# Promoting relaxation through essential oil-enhanced digital hypnotherapy: A randomized controlled trial

**DOI:** 10.1017/S0033291726103778

**Published:** 2026-03-30

**Authors:** Steven Ngandeu Schepanski, Martin Bogdanski, Katja Karolina Siegfried, Steffen Schulz, Judith Czakert, Farid Kandil, Michael Teut, Julia Siewert, Georg Seifert

**Affiliations:** 1 Charité – Universitätsmedizin Berlin, corporate member of Freie Universität Berlin and Humboldt-Universität zu Berlin, Charité Competence Center for Traditional and Integrative Medicine (CCCTIM), Berlin, Germany; 2 Charité – Universitätsmedizin Berlin, corporate member of Freie Universität Berlin and Humboldt-Universität zu Berlin, Department of Pediatrics, Division of Oncology and Hematology, Berlin, Germany; 3 Charité – Universitätsmedizin Berlin, corporate member of Freie Universität Berlin and Humboldt-Universität zu Berlin, Institute of Social Medicine, Epidemiology and Health Economics, Berlin, Germany; 4 Department of Internal Medicine and Nature-Based Therapies, Immanuel Hospital Berlin, Berlin, Germany; 5 Institute for Social Medicine & Epidemiology, Medizinische Hochschule Brandenburg Theodor Fontane, Hochstrasse 15, Brandenburg an der Havel, Germany

**Keywords:** aromatherapy, digital hypnotherapy, eHealth, essential oils, multisensory intervention, online mental health intervention, randomized controlled trial, relaxation, subjective calmness

## Abstract

**Background:**

Digital interventions for promoting relaxation are increasingly popular, yet few combine multiple sensory modalities. This study evaluated the effectiveness of a fully digital relaxation program combining hypnotherapy with aromatherapy and explored whether the scent can induce a conditioned relaxation effect.

**Methods:**

In this four-arm randomized controlled trial (*N* = 504), participants were assigned to one of four groups for a 4-week intervention: (a) combined (hypnotherapy + aromatherapy), (b) hypnotherapy-only, (c) aromatherapy-only, or (d) control (minimal intervention pause). Sessions were self-guided and delivered online every 2 days. The primary outcome was subjective calmness, assessed via the calmness–restlessness subscale of the Multidimensional Mood Questionnaire. Secondary outcomes included perceived stress (PSS-10) and well-being (WHO-5). A fifth week with aromatherapy-only exposure was conducted in the combined and aromatherapy-only groups to test for conditioning.

**Results:**

At post-intervention, both hypnotherapy-involved groups reported significant greater calmness than controls. The combined group showed a mean difference of *β* = 2.08 (95% CI: 0.50–3.65, *p* = 0.010, *d* = 0.38), while the hypnotherapy-only group showed *β* = 1.80 (95% CI: 0.24–3.37, *p* = 0.024, *d* = 0.33). Both effects were consistent across intention-to-treat and per-protocol analyses. Within-group improvements in calmness were also observed across all groups. No significant differences emerged from the conditioning test in week 5.

**Conclusions:**

Digital hypnotherapy improved relaxation, with modest added benefit from aromatherapy. The results support the use of multisensory digital tools to enhance subjective calmness. However, no evidence for conditioned effects of the scent was observed under the current conditions.

## Introduction

In recent years, stress and insufficient relaxation have markedly increased in industrialized societies (Piao, Xie, & Managi, 2024), particularly among students, young adults, and working individuals (McGinty, Presskreischer, Anderson, Han, & Barry, 2020; McGinty, Presskreischer, Han, & Barry, 2020; Wang et al., 2023). For instance, a large representative German survey from 2021 found that 64% of adults felt at least occasionally stressed, with frequent stress rising from 20% in 2013 to 26% in 2021 (Techniker Krankenkasse, 2021). While women continue to report high emotional exhaustion, stress levels have recently increased more strongly among men, affecting individuals across all working-age groups (Techniker Krankenkasse, 2021). Similar trends have been observed globally (Piao et al., 2024).

Chronic stress is a well-documented risk factor for anxiety (Hussenoeder et al., 2024), depressive symptoms (Tafet & Nemeroff, 2016), cardiovascular disease (Kivimaki & Steptoe, 2018; Osborne et al., 2020), and sleep disturbances (Han, Kim, & Shim, 2012; Yoo et al., 2023). Yet many individuals with elevated stress never seek formal treatment due to stigma, limited access, or time constraints (Andrade et al., 2014; Clement et al., 2015). This highlights the need for low-threshold, scalable, and evidence-based interventions to promote everyday relaxation and stress regulation.

Brief self-administered ‘microbreaks’ embedded into daily life are one promising approach. Microbreaks of 5–10 minutes can improve stress perception, emotional well-being, and cognitive performance (Albulescu et al., 2022; Kim, Park, & Niu, 2017; Zacher, Brailsford, & Parker, 2014), particularly when the break involves targeted strategies (Trougakos, Hideg, Cheng, & Beal, 2014). Despite these findings, few interventions are specifically designed to combine effectiveness, user-friendliness, and digital accessibility in such a short timeframe.

Hypnotherapy is a well-established psychological technique with demonstrated effects in both clinical and nonclinical populations (Häuser, Hagl, Schmierer, & Hansen, 2016; Milling, Valentine, LoStimolo, Nett, & McCarley, 2021). Meta-analyses and randomized controlled trials (RCTs) have shown that hypnotherapy can reduce symptoms of anxiety, depression, posttraumatic stress disorder (PTSD), as well as improve pain management, sleep quality, and stress regulation (Milling et al., 2021; Rotaru & Rusu, 2016; Valentine, Milling, Clark, & Moriarty, 2019; Yerzhan, Ayazbekova, Lavage, & Chelly, 2025). Typically delivered through verbal imagery and suggestions (Williamson, 2019), hypnotherapy promotes parasympathetic nervous system (PNS) activation and attentional control (Bicego, Rousseaux, Faymonville, Nyssen, & Vanhaudenhuyse, 2021; Jensen, Adachi, & Hakimian, 2015), making it well suited for stress-reduction goals (Fisch, Brinkhaus, & Teut, 2017; Fisch, Trivakovic-Thiel, et al., 2020b; Tanev & Daitch, 2025). Digitally guided hypnotherapy, for instance delivered via audio recordings, has shown promise in promoting relaxation and emotional balance (Fisch, Binting, et al., 2020a; Fuhr et al., 2021; Hauser, Hagl, Schmierer, & Hansen, 2016). However, even if a few studies rigorously evaluated its effectiveness in daily-life settings (Hauser et al., 2016; Sucala et al., 2013; Zhao, You, Shi, & Gan, 2015), its application in microbreak formats, particularly for use during daily life, remains underexplored.

In parallel, interest has grown in using olfactory cues to support relaxation (Shin, Bae, Afzal, Kondoh, & Lee, 2023). The olfactory system has privileged access to limbic structures, such as the amygdala and hippocampus (Salimi, Tabasi, Nazari, Ghazvineh, & Raoufy, 2022; Yang et al., 2025), allowing scents to evoke vivid emotional and autobiographical responses (Herz, 2004; Kontaris, East, & Wilson, 2020; Lopis, Valentin, & Manetta, 2023; Shepherd, 2003).

Experimental and clinical studies have shown that certain essential oils and their respective scents, such as lavender (*Lavandula angustifolia*) or bergamot (*Citrus bergamia*), are associated with reductions in perceived stress, physiological arousal, and improved mood (Czakert et al., 2024; Kritsidima, Newton, & Asimakopoulou, 2010; Rombola et al., 2017). Similarly, forest environments, particularly those rich in natural scents from trees and plants, such as volatile organic compounds emitted by conifers, have been associated with reductions in stress biomarkers and physiological arousal in studies on forest bathing (Kontaris et al., 2020; Siah et al., 2023). Moreover, odors paired with emotional states may later retrieve those states through classical conditioning (Herz, 2004; Herz & Engen, 1996).

Despite this neurobiological rationale, olfactory elements of essential oils are rarely integrated into digital self-administered tools. In fact, we are not aware of studies that have tested whether repeated pairing of essential oil-based scents with relaxation can produce conditioned responses in an online setting. According to classical conditioning theory, a neutral stimulus (scent) can elicit a learned response (relaxation) if paired frequently with an unconditioned stimulus (hypnotherapy) that naturally evokes the response (Zimbardo, Johnson, McCann, & Carter, 2003).

The present RCT tested a digital intervention that combined audio-guided hypnotherapy with a forest-scented essential oil-based spray, hereafter also referred to as aromatherapy. The primary objective was to evaluate whether the combined intervention improved subjective calmness more than a minimal intervention as control and whether this improvement is better compared to how hypnotherapy alone can improve calmness compared to the same control. A secondary aim was to assess whether the scent alone, after repeated pairing, could evoke a relaxation response, consistent with a conditioning mechanism.

## Methods

### Study design

**Figure 1.**
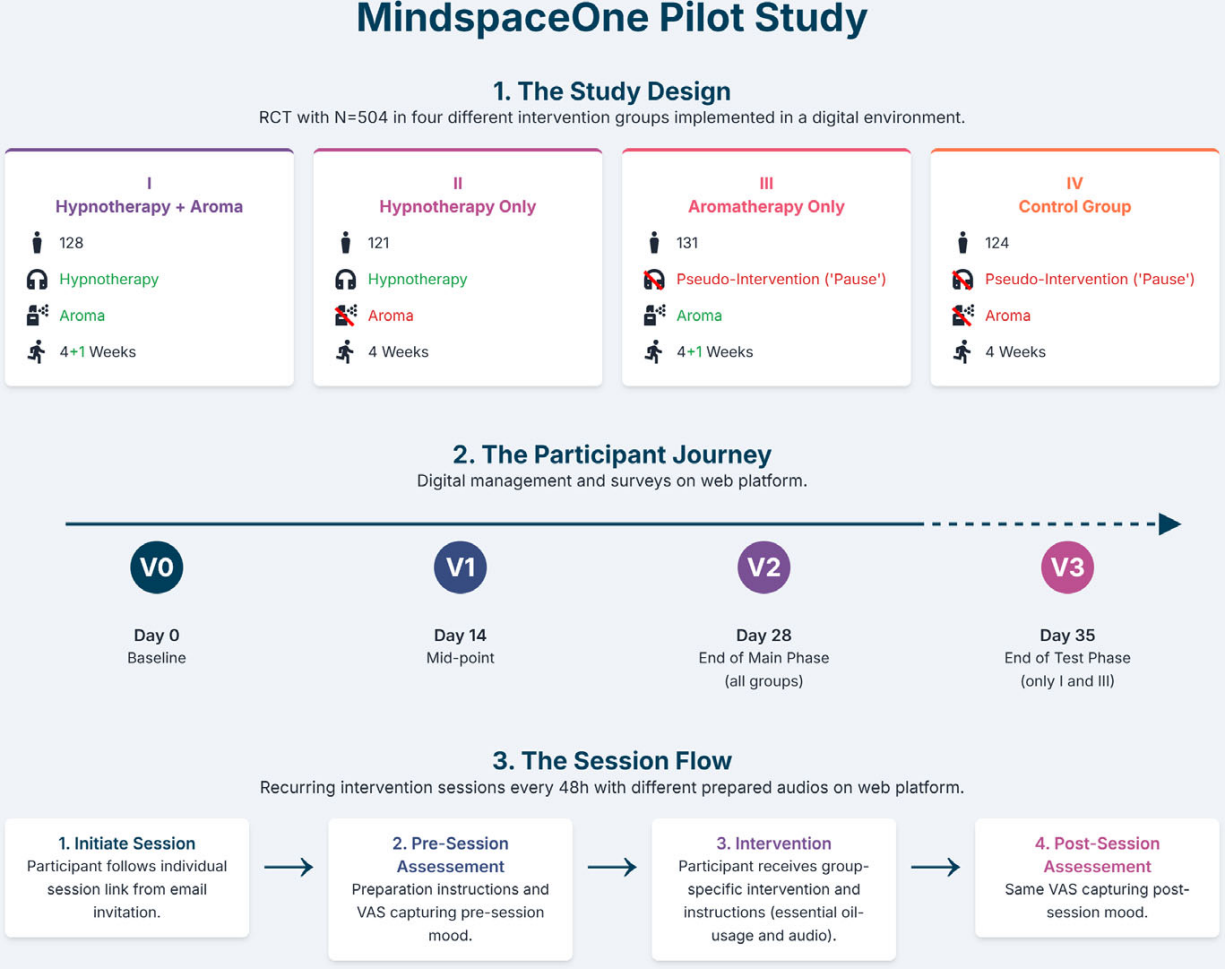
Study design and timeline of interventions and assessments.

This study implemented a four-arm, parallel-group RCT evaluating a digitally guided hypnotherapy intervention, with and without a physical olfactory cue through essential oils to promote subjective relaxation. The intervention was delivered via an automated web-based platform on an in-house instance of SoSci Survey (Version 3.5.02, SoSci Survey GmbH, Munich, Germany). Participants in all study arms received standardized instructions and engaged in their assigned intervention over a 4-week period, followed by a 1-week follow-up in selected groups (Figure 1). Intervention sessions were scheduled at fixed 48-hour intervals and participants received an automated invitation (via email) to each session via the study platform; no additional reminders were sent for session completion. Adherence was tracked digitally based on session access and completion logs. In contrast, assessment visits (V0–V3) were accompanied by automated reminders (email) if not completed within a predefined time window (V0 after 48 hours; V1–V3 after 24 hours), with no reminder sent if the visit was completed earlier. The trial was registered in the German Clinical Trials Register (Deutsches Register Klinischer Studien, DRKS; ID: DRKSS00034509) on June 24, 2023. The study protocol, including all methodological details, is available within the registry. All procedures were approved by the Institutional Review Board of Charité – Universitätsmedizin Berlin (Ethikkommission der Charité, Campus Charité Mitte, reference number EA4/086/23) and were conducted in accordance with the Declaration of Helsinki (1975, as revised in 2008) and relevant local data protection regulations.

### Participants

Adults aged 18–65 years, of any gender, residing in Germany, with internet access and German proficiency, were eligible. Exclusion criteria included self-reported current or past psychiatric history, concurrent psychotherapeutic/hypnotherapeutic treatment, or participation in overlapping clinical trials. Recruitment occurred via social media, online platforms, university channels, and public postings. Interested individuals consented and enrolled directly on the SoSci Survey platform, where they received detailed study information. Participants were allowed to withdraw from the intervention at any time without negative consequences. No prespecified stop rules were defined, as no pharmacological agents or invasive procedures were involved. Adherence was tracked digitally. Participants received three essential oil sprays as a token of appreciation upon study completion.

### Interventions

#### Rationale for essential oil selection

The forest-associated essential oil spray (*Pinus cembra*, ‘Swiss pine forest’) (Zirbenwald Raumspray bio, PRIMAVERA LIFE GMBH, art. no. 19514) was selected *a priori* to provide an ecologically coherent and broadly acceptable olfactory cue in a heterogeneous, nonclinical adult sample. Forest-related sensory cues are theoretically relevant to relaxation because olfactory inputs have direct access to limbic and paralimbic circuits implicated in affective processing, autobiographical memory, and stress regulation (Bratman et al., 2024; Shin et al., 2023). In addition, nature-associated stimuli are widely conceptualized as restorative, and evidence from research on nature exposure and forest bathing indicates that forest environments and their sensory characteristics are associated with reductions in subjective stress and physiological arousal (Queirolo et al., 2024; Siah et al., 2023; Simpattanawong, Li, & McEwan, 2024). Together, these considerations support the plausibility of using a forest-congruent odor as a candidate cue for downregulation and potential associative learning in a digital relaxation context. Detailed information about the ingredients, composition, and characteristics of the essential oil spray used are reported in accordance with the Transparent Reporting for Essential oil & Aromatic Therapeutic Studies (TREATS) checklist (Reven et al., 2024) (Supplementary Material 1). We additionally completed the TREATS checklist for the aromatherapy component (total score 20/30, inhalation pathway, Supplementary Material 2).

#### Combined group (digitally guided hypnotherapy and olfactory cue/aromatherapy)

Participants in this group received a combination of digitally guided hypnotherapy and a physical olfactory cue through essential oils. This group is hereafter referred to as MindSpaceOne, reflecting the acronym of the study title: *Multimodal Intervention for Neurophysiological Downregulation via Scent and Psychological Affect Conditioning Elements – Online Neurocognitive Evaluation.* At the start of each session, they were instructed to spray the essential oil once or twice into the surrounding air and then begin the audio-guided session.

The audio-based hypnotherapy was developed and narrated by a clinical hypnotherapist (M.T.) (Supplementary Material 3). The first session lasted approximately 22 minutes and served as an introduction, followed by a shorter 11-minute ‘refresher’ session on subsequent interventions. The script included verbal suggestions to support PNS activation through slowed breathing, guided inner imagery, and metaphorical suggestions for emotional release. The olfactory cue from the essential oil spray was interwoven into the script through suggestions to associate the scent with calmness and inner balance.

The intended conditioning followed a classical conditioning rationale: hypnotherapy served as the unconditioned stimulus (US) that elicited a relaxation response (unconditioned response, UR), while the essential oil-based scent, initially a neutral stimulus, was repeatedly paired with this state and intended to become a conditioned stimulus (CS). Over time, the scent alone was hypothesized to elicit a conditioned relaxation response (CR).

#### Hypnotherapy-only group (digitally guided hypnotherapy)

This group received the same audio content and schedule as the MindSpaceOne group, including one 22-minute introductory session and repeated 11-minute sessions thereafter but without the use of any scents (Supplementary Material 3).

#### Aromatherapy-only group (olfactory cue and pause)

Participants in this group were instructed to spray the same essential oil product as used in the MindSpaceOne group once or twice into the surrounding air before beginning a brief 5-minute audio minimal intervention. The audio gently invited them to settle into a comfortable posture, close their eyes if desired, and take time to consciously experience the scent. No further guidance was provided because this group was supposed to test the standalone effect of aromatherapy with the olfactory cue, without hypnotherapy (Supplementary Material 3).

#### Control group (minimal-intervention pause)

The control group listened to the same 5-minute audio as used in the aromatherapy-only group, but without any olfactory component. The audio invited participants to take a short minimal intervention pause, make themselves comfortable, and ‘do nothing’ for a few minutes before gently concluding. No specific suggestions for relaxation or sensory focus were included (Supplementary Material 3).

#### Follow-up phase (week 5)

During the 1-week follow-up, only the MindSpaceOne and aromatherapy-only groups received additional sessions. Both groups were instructed to spray the essential oil into the room as before and listen to the same 5-minute audio guide used in the aromatherapy and control groups. This served to test whether the scent of the essential oil alone, after prior repeated pairing with hypnotherapy, could elicit a conditioned relaxation response within the MindSpaceOne group.

### Outcomes

The primary outcome was subjective calmness measured using the calmness-restlessness subscale of the Multidimensional Mood Questionnaire (MDMQ) (Steyer, Schwenkmezger, Notz, & Eid, 1994). The MDMQ is a validated self-reported instrument comprising three bipolar subscales, namely calmness-restlessness, alertness-tiredness, and good mood-bad mood. Each subscale contains four adjective pairs rated on a 5-point Likert scale, with higher scores reflecting a more positive affective state. The MDMQ has demonstrated good internal consistency in prior research, with Cronbach’s α between 0.86 and 0.94, depending on the population and setting (Steyer et al., 1994). The other two subscales, alertness-tiredness and good mood-bad mood, along with the MDMQ total score, were examined as secondary outcomes.

Further secondary outcomes included the Perceived Stress Scale, 10-item version (PSS-10) (Cohen, Kamarck, & Mermelstein, 1983; Klein et al., 2016), which assesses perceived stress over the preceding 2 weeks using 10 items rated on a 5-point scale. The PSS-10 yields a total score and two subscales, namely helplessness and self-efficacy, showing robust psychometric properties with a good internal consistency (Cronbach’s *α* = 0.84) (Klein et al., 2016). Also, the World Health Organization Five Well-Being Index (WHO-5) (Bech, Olsen, Kjoller, & Rasmussen, 2003), a five-item measure of current psychological well-being was integrated. Each item is rated on a 6-point scale, with the total score ranging from 0 to 25. Raw scores below 13 are indicative of poor mental well-being. The WHO-5 has shown excellent internal consistency (Cronbach’s *α* > 0.89) (Bech et al., 2003; Bonsignore, Barkow, Jessen, & Heun, 2001).

Harms and adverse events were monitored via self-report throughout the study. Participants were instructed to report any discomfort, unexpected negative emotional reactions, or other adverse events through the SoSci platform or by contacting the study team directly. Given the low-risk nature of both hypnotherapeutic audio sessions and the aromatherapy sprays, no serious adverse events were anticipated.

### Randomization, allocation, and blinding

Randomization was performed via an automated procedure embedded in the SoSci Survey platform. During registration and a prebaseline sociodemographic survey, participants were randomly assigned (1:1:1:1) to one of the four study arms (MindSpaceOne, hypnotherapy-only, aromatherapy-only, and control) using SoSci’s internal randomization function.

Participants were partially blinded as follows: Consent materials stated a random assignment to one of four groups differing in features such as the use of an essential oil spray and involving hypnotherapy. Due to the sensory nature of the intervention, complete blinding was not feasible. It can be assumed that the use or absence of a spray was quickly noticed. However, nothing was revealed about the nature and specific content of the hypnotherapy audio. Furthermore, participants were unaware of the conditioning background or comparative aims, limiting expectancy effects and reducing contamination risk in this unsupervised, digital context.

### Sample size calculation

Sample size was based on detecting a small-to-medium effect (Cohen’s *d* = 0.35) for primary comparison between the MindSpaceOne and control groups. This assumption reflected the lack of prior studies on combined digital hypnotherapy and aromatherapy interventions.

Using a one-sided *α* = 0.05 and 80% power (*β* = 0.20), the required sample size was 102 participants per group. To accommodate an expected 20% dropout rate, the target sample size was increased to *n* = 500 participants across all arms. This oversampling aimed to preserve analytical power despite dropout over the 4-week intervention period or at follow-up period.

### Data analysis

#### Primary and secondary analysis

Between-group differences in subjective calmness at the postintervention (V2) were assessed using analysis of covariance (ANCOVA), controlling for baseline calmness (V0). Within-group changes from V0 to V2 were additionally evaluated via one-sample *t* tests on individual change scores, performed separately for each group. All analyses were conducted in both the intention-to-treat (ITT) and per-protocol (PP) populations.

Secondary outcomes (remaining MDMQ subscales, PSS-10, WHO-5) were analyzed using the same Analysis of Covariance (ANCOVA) framework, with post-intervention scores as outcomes, group allocation as predictor, and baseline scores as covariates. Pairwise comparisons were performed for MindSpaceOne and hypnotherapy-only groups, each compared to the control condition.

To assess whether aromatherapy alone could elicit a conditioned relaxation response, calmness scores at follow-up (V3) were compared between the MindSpaceOne and aromatherapy-only groups, controlling for V2 calmness.

#### Exploratory analysis

We explored whether olfactory preference moderated intervention effects. Participants rated the aromatherapy scent as negative, neutral, or positive at the study end. ANCOVA models were repeated in the subgroup with neutral or positive essential oil ratings, given known valence effects on affect and memory (Chu & Downes, 2002; Herz, 2004; Herz & Schooler, 2002).

#### Handling of missing data

Missing data in the ITT sample were imputed using the Multivariate Imputation by Chained Equations (MICE) algorithm (Van Buuren & Groothuis-Oudshoorn, 2011) under the missing at random (MAR) assumption. Fifty datasets were imputed and pooled using Rubin’s rules.

#### Multiple testing correction

To control the false discovery rate (FDR) across all pairwise comparisons (*N* = 16), we used the Benjamini-Hochberg procedure with *Q* = 0.05 (Benjamini & Hochberg, 1995), as implemented in R via ‘*p.*adjust (method = “fdr”)’.

#### Adverse event classification

Adverse events were categorized into eight domains via rule-based keyword matching (e.g. ‘intervention-related’, ‘technical’). Classification was implemented in R using ‘stringr’ and ‘dplyr’.

#### Statistical software

All statistical analyses were performed using R (version 4.3.2). Key packages included ‘mice’ (imputation), ‘car’ (model diagnostics), ‘stats’ and ‘emmeans’ (ANCOVA and contrasts), ‘effectsize’ (Cohen’s *d*, 95% CIs), and ‘ggplot2’ (visualization). All significance tests were performed using an α = 0.05, and effect sizes were reported using Cohen’s *d* with corresponding 95% CIs for all group comparisons.

## Results

### Participants

**Figure 2.**
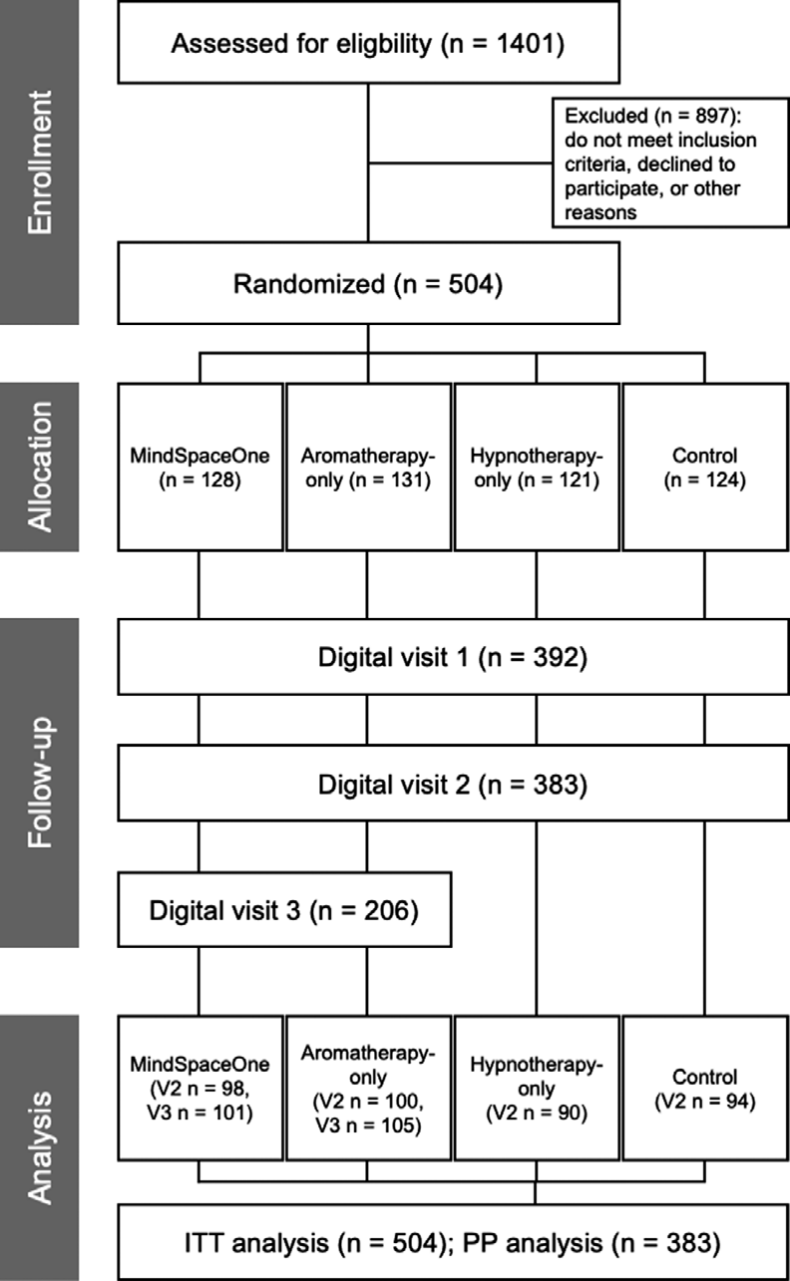
CONSOFRT flow diagram. Flow of participants through the study, from initial enrollment and randomization to completion of digital assessments. All groups completed assessments at baseline (V0), week 2 (V1), and week 4 (V2). The MindSpaceOne and aromatherapy-only groups completed an additional follow-up at week 5 (V3). Final sample sizes at each wave reflect participant retention and assessment completion. The intention-to-treat (ITT) population consisted of *n* = 504 and the per-protocol (PP) population of *n* = 383 participants.

A total of 1401 individuals accessed the study platform; 504 met eligibility criteria and were randomized to MindSpaceOne (*n* = 128), aromatherapy-only (*n* = 131), hypnotherapy-only (*n* = 121), or control (*n* = 124), constituting the ITT sample (Figure 2). Although registration was capped at 500, four additional participants were likely enrolled due to simultaneous sever requests during consent.

Baseline assessment (V0) was completed by 485 participants. Follow-ups were completed by 392 participants at week 2 (V1) and 383 participants at week 4 (V2), marking the final time point for the hypnotherapy-only and control groups. Finally, 206 participants in the MindSpaceOne and aromatherapy-only conditions completed the follow-up at week 5 (V3).

The PP sample for the core intervention period (V0–V2) included 383 participants. Dropout from V0 to V2 was 21.03%, with most dropouts occurring between V0 and V1 (19.2%) and minimal loss from V1 to V2 (2.3%). Retention from V0 was 80.8% at V1 and 79.0% at V2, indicating a strong participant engagement after initial follow-up. Assessment visits were accompanied by automated reminders as described in the Methods, whereas intervention sessions were invitation based without additional reminders.

### Study characteristics

Groups were well balanced in gender, age, education, and employment (Table 1). Most participants identified as female (82–86%) and were between 26 and 45 years. Over half held a university degree, and around three quarters were employed.

**Table 1.** Sociodemographic characteristics, stress-related background, and prior use of relaxation techniques, by group

Variable	MindSpaceOne	Hypnotherapy-only	Aromatherapy-only	Control
*n*	128	121	131	124
Gender, *n*				
Female, *n* (%)	109 (85.2)	100 (82.6)	108 (82.4)	106 (85.5)
Male, *n* (%)	19 (14.8)	21 (17.4)	23 (17.6)	18 (14.5)
Other, *n* (%)	0 (0)	0 (0)	0 (0)	0 (0)
Age classes, years				
18–25, *n* (%)	9 (7.03)	12 (9.92)	16 (12.2)	21 (16.9)
26–35, *n* (%)	51 (39.8)	39 (32.2)	43 (32.8)	35 (28.2)
36–45, *n* (%)	27 (21.1)	23 (19.0)	37 (28.2)	29 (23.4)
46–55, *n* (%)	22 (17.2)	21 (17.4)	20 (15.3)	21 (16.9)
56–65, *n* (%)	19 (14.8)	26 (21.5)	15 (11.5)	18 (14.5)
Education, degree				
University degree, *n* (%)	68 (53.1)	68 (56.2)	68 (51.9)	74 (59.7)
Apprenticeship, *n* (%)	46 (35.9)	39 (32.2)	49 (37.4)	38 (30.6)
Other degree, *n* (%)	6 (4.69)	6 (4.96)	5 (3.82)	4 (3.23)
No degree, *n* (%)	8 (6.25)	8 (6.61)	9 (6.87)	8 (6.45)
Income, EUR				
3000<, *n* (%)	37 (17.6)	31 (16.4)	36 (16.1)	32 (17.2)
2000–2999, *n* (%)	41 (19.5)	42 (22.2)	50 (22.4)	46 (24.7)
1500–1999, *n* (%)	16 (7.62)	19 (10.1)	13 (5.83)	9 (4.84)
1000–1499, *n* (%)	17 (8.10)	10 (5.29)	9 (4.04)	13 (6.99)
<1000, *n* (%)	3 (1.43)	9 (4.76)	10 (4.48)	9 (4.84)
I prefer not to answer, *n* (%)	7 (3.34)	8 (4.23)	10 (4.48)	8 (4.30)
No income, *n* (%)	7 (3.33)	2 (1.06)	3 (1.35)	7 (3.76)
Occupation, *n*				
Employee, *n* (%)	94 (73.4)	93 (76.9)	99 (75.6)	87 (70.2)
Civil servant, *n* (%)	6 (4.69)	4 (3.31)	3 (2.29)	8 (6.45)
Self-employed, *n* (%)	5 (3.91)	4 (3.31)	9 (6.87)	7 (5.65)
Retired, *n* (%)	1 (0.78)	3 (2.48)	3 (2.29)	1 (0.81)
Student, *n* (%)	12 (9.38)	10 (8.26)	12 (9.16)	19 (14.8)
Jobless, *n* (%)	7 (5.47)	1 (0.83)	2 (1.53)	1 (0.81)
Other, *n* (%)	3 (2.34)	5 (4.13)	3 (2.29)	5 (4.03)
NA, *n* (%)	0 (0)	1 (0.83)	0 (0)	0 (0)
Perceived stressors^a^, *n*				
Children (0–6 years), *n* (%)	17 (8.10)	19 (10.1)	23 (10.3)	20 (10.8)
Children (7–13 years), *n* (%)	13 (6.19)	21 (11.1)	20 (8.97)	15 (8.06)
Children (14 < years), *n* (%)	10 (4.76)	11 (5.82)	16 (7.17)	10 (5.38)
Handicapped child, *n* (%)	1 (0.48)	3 (1.59)	1 (0.45)	2 (1.08)
Handicapped adult, *n* (%)	16 (7.62)	13 (6.88)	14 (6.28)	17 (9.14)
Night shifts, *n* (%)	13 (6.19)	13 (6.88)	14 (6.28)	11 (5.91)
Physical work, *n* (%)	11 (5.24)	14 (7.41)	16 (7.17)	21 (11.3)
Seated and stood work, *n* (%)	60 (28.6)	63 (33.3)	58 (26.0)	56 (30.1)
Shifting shifts, *n* (%)	18 (8.57)	24 (12.7)	24 (10.8)	25 (13.4)
Seated work, *n* (%)	59 (28.1)	47 (24.9)	51 (22.9)	52 (28.0)
Weekend work, *n* (%)	26 (12.4)	26 (13.8)	39 (17.5)	27 (14.5)
Used relaxation methods^a^, *n*				
Sport, *n* (%)	71 (33.8)	53 (28.0)	71 (31.8)	73 (39.2)
Yoga, *n* (%)	42 (20.0)	37 (19.6)	40 (17.9)	29 (25.6)
Meditation, *n* (%)	27 (12.9)	22 (11.6)	23 (10.3)	22 (11.8)
PMR, *n* (%)	8 (3.81)	14 (7.41)	6 (2.69)	6 (3.23)
Massage, *n* (%)	18 (8.57)	13 (6.88)	26 (11.7)	16 (8.60)
Hypnosis, *n* (%)	1 (0.48)	2 (1.06)	1 (0.45)	0 (0)
Resting, *n* (%)	13 (6.19)	10 (5.29)	14 (6.28)	8 (4.30)
Breathing exercise, *n* (%)	23 (11.0)	26 (13.8)	31 (13.9)	25 (13.4)
Autogenic training, *n* (%)	3 (1.43)	8 (4.23)	6 (2.69)	4 (2.15)
Aromatherapy, *n* (%)	4 (1.90)	4 (2.12)	5 (2.24)	3 (1.61)

PMR, progressive muscle relaxation.

a Multiple answers per person allowed.

Prior use of relaxation was common, especially sports, breathing exercises, and yoga. Massage was more frequently in the aromatherapy-only group (11.7%), and progressive muscle relaxation (PMR) and autogenic training were slightly more common in the hypnotherapy-only group. Hypnosis and aromatherapy were rare across all groups.

### Primary outcome: subjective calmness

Subjective calmness was assessed via the calmness-restlessness subscale of the MDMQ. At baseline (V0), group means ranged from 21.81 (SD = 6.14; hypnotherapy-only) to 24.11 (SD = 7.27; MindSpaceOne), with the control group at 23.15 (SD = 6.53), suggesting moderate stress levels across all groups (Supplementary Material 4). After the 4-week intervention (V2), all groups reported increased calmness, with the largest gains observed in the two hypnotherapy-involved conditions. The MindSpaceOne group reached a mean calmness-restlessness score of 28.96 (SD = 6.07), compared to 28.02 (SD = 5.53) in the hypnotherapy-only group, and 26.16 (SD = 5.96) in the control group.

ANCOVA controlling for baseline scores confirmed that both MindSpaceOne (*β* = 2.08, 95% CI: 0.50–3.65, *p* = 0.010, adjusted *p* = 0.044, *d* = 0.38) and hypnotherapy-only (*β* = 1.80, 95% CI: 0.24–3.37, *p* = 0.024, adjusted *p* = 0.064, *d* = 0.33) significantly outperformed the control group (Table 2). Within-group analyses showed statistically significant improvements in all arms. The MindSpaceOne group improved by 4.54 points (95% CI: 3.10–5.98), the hypnotherapy-only group by 5.15 points (95% CI: 3.72–6.57), and the control group by 3.11 points (95% CI: 1.89–4.33), all *p*s < 0.001 (Supplementary Material 5).

**Table 2.** Primary and secondary outcomes (ITT, *n* = 504)

Outcome	Estimate (*β*)	95% CI		*p*	Adjusted *p*	Cohen’s *d*
		Lower	Upper			
***Primary outcome: MDMQ calmness-restlessness***						
MindSpaceOne vs. control	2.076	0.50	3.65	**0.010**	**0.044**	0.38
Hypnotherapy-only vs. control	1.803	0.24	3.37	**0.024**	0.064	0.33
***Secondary outcomes***						
**MDMQ alertness-tiredness**						
MindSpaceOne vs. control	2.345	0.83	3.86	**0.002**	**0.032**	0.44
Hypnotherapy-only vs. control	2.070	0.47	3.67	**0.011**	**0.044**	0.39
**MDMQ good mood-bad mood**						
MindSpaceOne vs. control	0.928	-0.42	2.28	0.178	0.316	0.20
Hypnotherapy-only vs. control	0.643	-0.74	2.03	0.364	0.485	0.14
**MDMQ total**						
MindSpaceOne vs. control	5.286	1.51	9.06	**0.006**	**0.044**	0.39
Hypnotherapy-only vs. control	4.521	0.67	8.37	**0.022**	0.064	0.33
**PSS-10 total**						
MindSpaceOne vs. control	-0.367	-1.78	1.05	0.612	0.699	0.07
Hypnotherapy-only vs. control	-0.714	-2.22	0.80	0.354	0.485	0.14
**PSS-10 helplessness**						
MindSpaceOne vs. control	-0.416	-1.46	0.63	0.437	0.538	0.11
Hypnotherapy-only vs. control	-0.668	-1.74	0.41	0.223	0.357	0.18
**PSS-10 self-efficacy**						
MindSpaceOne vs. control	-0.035	-0.74	0.67	0.923	0.923	0.01
Hypnotherapy-only vs. control	-0.087	-0.87	0.70	0.829	0.884	0.03
**WHO 5**						
MindSpaceOne vs. control	-1.227	-2.37	-0.09	**0.035**	0.080	0.30
Hypnotherapy-only vs. control	-1.007	-2.22	0.21	0.105	0.210	0.25

CI, confidence interval; ITT, intention-to-treat; MDMQ, Multidimensional Mood State Questionnaire; PSS-10, Perceived Stress Scale 10 items. Bold *p* values indicate *p* < 0.05.

To assess clinical relevance, we applied two complementary approaches. First, the MindSpaceOne-control group difference of 2.8 points exceeds the commonly used minimal important difference of 0.5 SD (approximately 1.6 points; Hinz, Daig, Petrowski, & Brähler, 2012). Second, the difference corresponds to an average shift of 0.35 points per item on the 8–40 scale, indicating participants felt about one third of a response category calmer per item.

In the PP analysis (Supplementary Material 6), the MindSpaceOne (*β* = 2.59, 95% CI: 1.08–4.10, *p* = 0.0009, adjusted *p* = 0.007, *d* = 0.49) and hypnotherapy-only results (*β* = 2.25, 95% CI: 0.71–3.79, *p* = 0.005, adjusted *p* = 0.016, *d* = 0.43) supported ITT findings. The consistent effects across ITT and PP analyses indicate a robust impact of the interventions on subjective relaxation.

### Secondary outcomes

#### Mood dimensions

In the ITT analysis, both hypnotherapy-based groups reported significantly higher alertness at postintervention compared to controls (MindSpaceOne: *β* = 2.35, 95% CI: 0.83 to 3.86, *p* = 0.002, adjusted *p* = 0.032, *d* = 0.44; hypnotherapy-only: *β* = 2.07, 95% CI: 0.47 to 3.67, *p* = 0.011, adjusted *p* = 0.044, *d* = 0.39) (Table 2). Similarly, MDMQ total scores were higher in both groups (MindSpaceOne: *β* = 5.29, 95% CI: 1.51 to 9.06, *p* = 0.006, adjusted *p* = 0.044, *d* = 0.39; hypnotherapy-only: *β* = 4.52, 95% CI: 0.67 to 8.37, *p* = 0.022, adjusted *p* = 0.064, *d* = 0.33) (Table 2). No significant between-group differences emerged for the good mood–bad mood subscale. Similar results were observed in the PP population (Supplementary Material 6).

#### Perceived stress

Across all groups, perceived stress (PSS total) decreased slightly from baseline to V2, but these changes did not significantly differ between groups (all adjusted *p*s > 0.20; Table 2 and Supplementary Material 6).

#### Psychological well-being

Participants in the MindSpaceOne group reported significantly lower WHO-5 well-being scores at postintervention, meaning higher well-being, compared to the control group (*β* = -1.23, 95% CI: -2.37 to -0.09, *p* = 0.035, adjusted *p* = 0.080, *d* = 0.30) (Table 2). While this effect was only marginally significant after correction, it was replicated in the PP analysis (*β* = -1.49, 95% CI: -2.62 to -0.36, *p* = 0.01, adjusted *p* = 0.023, *d* = 0.38) (Supplementary Material 6). The hypnotherapy-only group showed a similar result (*β* = -1.09, 95% CI: -2.25 to 0.06, *p* = 0.064, adjusted *p* = 0.119, *d* = 0.28), but this did not reach statistical significance. This translates to slightly but significantly improved well-being exclusively in the MindSpaceOne group.

### Conditioning effects

No significant differences in subjective calmness were found between MindSpaceOne and aromatherapy-only groups at the follow-up time point (V3), either in ITT or PP analyses (Supplementary Material 7). This provides no evidence for a conditioned relaxation response elicited by the scent alone.

### Exploratory analysis: Olfactory preference subgroups

To explore whether olfactory preferences influenced the potential conditioning effect, we conducted a stratified PP analysis, based on an optional question about the scent preferences at V0, including only those participants who rated the scent as pleasant or neutral at baseline (*n* = 194). Individuals who reported disliking the scent (*n* = 7) or did not provide a rating (*n* = 3) were excluded. Among participants with neutral or positive scent ratings, group differences at V3 remained nonsignificant (*β* = -0.433, 95% CI: -1.94 to 1.07, *p* = 0.42, *d* = 0.12) (Supplementary Material 8), suggesting olfactory preference did not moderate conditioning effects.

### Intervention fidelity and adverse events

Adherence was generally high across groups visualized by a session-level heatmap (Supplementary Material 9), showing completion status across time points and groups. Participants in the aromatherapy-only group completed the most sessions on average (12.3/18), followed by the MindSpaceOne (10.7/18), control (9.4/14), and hypnotherapy-only groups (9.2/14).

Self-reported adverse events were infrequent and occurred across all groups. At V1, between 1 and 8 participants per group reported events; at V2, between 5 and 10; and at V3, 10 (aromatherapy-only) and 8 (MindSpaceOne) participants. All adverse event free-text responses (*n* = 60) were categorized using a keyword-based classification system (Supplementary Material 10). Most entries referred to external life events (*n* = 19) or physical complaints (*n* = 13), such as infections, pregnancy, or child-related sleep disruption, thus none of these were attributable to the intervention.

Only two individuals described negative reactions (Supplementary Material 10) plausibly linked to the intervention (e.g. frustration with repeated audio instructions or discomfort with the minimal intervention control group task). Other responses referenced psychological burden or implementation issues (e.g. lack of time). A few participants reported heightened emotional awareness, ambiguous effects, or minor confusion about session structure. No serious adverse events were reported and no participant withdrew due to intervention-related harm.

## Discussion

This RCT evaluated a digital relaxation intervention (MindSpaceOne) combining hypnotherapy and aromatherapy to promote calmness. Findings highlight the potential of scalable, low-threshold tools to enhance momentary well-being. Participants in the combined intervention reported significantly greater relaxation than controls, suggesting effectiveness even in unsupervised settings. Although no clinical threshold was predefined, the effect exceeded common benchmarks for meaningful change (SD = 0.5). Given participants’ daily stress exposures, modest improvements may reflect relevant functional gains.

The hypnotherapy-only group also reported substantial improvements, underscoring the central role of hypnotic techniques, likely via attentional, interoceptive, and autonomic modulation (De Benedittis, 2024; Diolaiuti, Huber, Ciaramella, Santarcangelo, & Sebastiani, 2019; Zhang et al., 2024). While aromatherapy added a numerical increase in calmness compared to hypnotherapy-only, overlapping confidence intervals suggest only a modest incremental benefit, possibly due to ceiling effects or limited personalized essential oil selection. Refinements like tailored scent selection or emotionally salient pairings may enhance impact. Interestingly, the control group also improved, indicating that structured self-care, such as a brief daily pause, can promote relaxation, aligning with findings on microbreaks (Fritz, Lam, & Spreitzer, 2011; Kim et al., 2017).

A central aim was to assess whether aromatherapy, when paired with hypnotherapy, could become a scent-conditioned trigger. No such effect emerged, possibly due to limited pairings (Dunsmoor, Bandettini, & Knight, 2007), inconsistent unconditioned responses (Miller, Barnet, & Grahame, 1995), or insufficient emotional salience (LeDoux, 2013). While the forest scent was ecologically coherent and known to reduce stress and cortisol (Li, 2010; Park et al., 2009), it lacked personalization. Forest-based essential oil (blends) may activate limbic structures and promote parasympathic activity (Herz, 2009; Kiecolt-Glaser et al., 2008). Given their potential link to autobiographical memories and emotional states (Simon Chu & John J Downes, 2002; Herz, 2004; Willander & Larsson, 2007; Yamamoto, Yokomitsu, & Kobayashi, 2022), future studies should explore individualized scent selection and contextual variability.

Our findings contribute to the growing field of digital microinterventions, which are brief, targeted tools for flexible, self-directed use in daily routines (Arigo, Schumacher, Baga, & Mogle, 2024; Baumel, Fleming, & Schueller, 2020). High adherence and minimal adverse events support the feasibility of MindSpaceOne. Delivered entirely online, the intervention may address care barriers for individuals with elevated stress (Borghouts et al., 2021).

Minor technical disruptions, namely delayed reminders and platform issues occurred but were resolved promptly. These events did not impact data integrity or safety but emphasize the need for robust infrastructure and user support when scaling digital interventions.

Several design aspects warrant consideration. First, the minimal intervention control condition did not fully account for nonspecific effects. One participant reported feeling ‘totally relaxed’ from the pause alone, suggesting that simple disengagement strategies can be effective. However, multiple arms and baseline-adjusted analyses strengthen interpretability. Second, reliance on self-reports limits mechanistic insight. Future research should include physiological measures (e.g. heart rate variability, salivary cortisol). Third, olfactory perception is highly individualized and though the forest essential oil had broad appeal, personal relevance likely modulates effectiveness (Herz, Eliassen, Beland, & Souza, 2004; Herz & Schooler, 2002; Saive, Royet, & Plailly, 2014). Future studies should examine personalized scent delivery, especially in clinical populations.

Our results align with prior studies on digital hypnotherapy (Lang et al., 2021; Scheffrahn, Hall, Muniz, & Elkins, 2025; Wang et al., 2025). Meta-analyses confirm benefits for stress, sleep, and well-being, even in brief, self-administered formats (Bissonnette et al., 2024; Eason & Parris, 2019; Rosendahl, Alldredge, & Haddenhorst, 2023). While multisensory hypnotherapy remains in its infancy, recent work combining suggestions with visual or tactile cues shows promise (Huntley, Nguyen, Albrecht, & Marinovic, 2024; Markmann et al., 2023; Mioli et al., 2021). The olfactory system’s direct links to memory and emotion-related brain regions (Chen, Kostka, Bitzenhofer, & Hanganu-Opatz, 2023; Ferdowsi et al., 2025; Kostka & Hanganu-Opatz, 2023) highlights its potential, but likely require tailored implementation.

MindSpaceOne exemplifies how evidence-informed digital tools can be embedded into daily life to support mental well-being. While this study found no clear evidence for olfactory-conditioned effects, it lays a foundation for refining stimulus delivery and pairing strategies. The brief, flexible format has broad applicability, from work breaks to academic stress, and may be especially useful for individuals with high stress or limited care access. Optimizing delivery of the scent, such as using more standardized and low-intensity delivery formats (e.g. aroma inhalers allowing brief, intermittent exposure) and tailoring olfactory stimuli to user preferences might enhance engagement and effectiveness. Inhaler-based delivery may be particularly suitable for digital interventions, as it could reduce variability related to ambient diffusion while facilitating personalized scent selection.

In sum, this study supports the potential of brief, low-threshold digital interventions to enhance subjective calmness. Even modest tools, when grounded in psychological and sensory science, can yield meaningful effects. While aromatherapy’s added value appears limited under current conditions, the broader sensory integration approach remains promising and merits further investigation.

## Supporting information

10.1017/S0033291726103778.sm001 Ngandeu Schepanski et al. supplementary material 1 Ngandeu Schepanski et al. supplementary material

10.1017/S0033291726103778.sm002 Ngandeu Schepanski et al. supplementary material 2 Ngandeu Schepanski et al. supplementary material

## Data Availability

All analyses related to this study are documented and fully reproducible via the following GitHub repository: https://github.com/sschepanski/MindSpaceOne. The repository includes the analysis scripts, statistical models, and visualization code. The anonymized dataset generated and analyzed during the current study will also be made available in the repository and can be freely accessed, reviewed, and downloaded by interested researchers. No personally identifiable information will be included in the shared dataset. Further inquiries can be directed to the corresponding author.

## Supplementary material

The supplementary material for this article can be found at http://doi.org/10.1017/S0033291726103778.
