# Supplementary material for: Promoting relaxation through essential oil-enhanced digital hypnotherapy: A randomized controlled trial
Source: Psychol Med. 2026 Mar 30;56:e80. doi: 10.1017/S0033291726103778 (PMC13079237; doi:10.1017/S0033291726103778)
Supplement: Ngandeu Schepanski et al. supplementary material 1 — Ngandeu Schepanski et al. supplementary material [file S0033291726103778sup001.docx]

# Online Supplementary Material

| **Supplementary Material 1. Characteristics about the used essential oil product.** | | | |
| --- | --- | --- | --- |
| **Product** | **Ingredients** | **Information about the underlying essential oil** | **Gas chromatography of the underlying essential oil** |
| **Original name**  Swiss Pine Forest Organic Spray [German: Zirbenwald Raumspray bio] | **Essential oil (Pinus Cembra):** 2.5-10%  **Ethanol:** 50-100% | **Essential oil botanical name:**  Pinus Cembra  **Country of Origin:**  Austria  **Country of Production:**  Germany  **Production Method:**  Essential oil obtained by steam distillation the twigs of Pinus Cembra  **Cultivation Method:**  Certified organic cultivation | b-Pinene = 42.81%  b-Myrcene = 2.31%  d-3-Carene = 0.01%  a-Terpinene = 11.46%  Limonene = 1.43%  b-Phellandrene = 1.31%  Terpinolene = 0.11%  Linalool = 12.79%  Camphor = 17.86%  a-Terpineol = 0.45%  Citronellol = 0.01%  t-b-Caryophyllene = 0.13%  Germacrene D = 0.38% |
| **Company**  PRIMAVERA Life GmbH |  |  |  |
| **Batch number**  A3514(3) |  |  |  |

**Supplementary Material 2**. TREATS Quality Appraisal Checklist is provided as a separate file.

| **Supplementary Material 3. Audio Script Transcripts** |
| --- |
| **Hypnosis Session 1 Script** |
| **Used in:** MindSpaceOne Group (Session 1), Hypnotherapy-Only Group (Session 1) |
| **Note:** In the MindSpaceOne Group, minor wording adjustments were made to include the olfactory cue, and these differences were indicated below using squared brackets. The text was translated from German into English for the purpose of the publication. |
| *Welcome to today's self-hypnosis session.*  *Make yourself comfortable in a quiet and pleasant place where you can let yourself be carried inwards for a while. [Allow yourself to perceive the pleasant aromatic scent of calm and serenity that surrounds you ... enjoy it].*  *The aim of this hypnosis is for you to allow yourself to be carried into a pleasant state of calm and serenity.*  *You can allow yourself to utilize the unconscious knowledge of your body, the calm reaction of your parasympathetic nervous system, by simply allowing what your unconscious, your body, has known for a long time, to happen. The parasympathetic nervous system is the part of our unconscious nervous system that carries us into rest and relaxation, while its counterpart, the sympathetic nervous system, activates us in everyday life.*  *Many people today are often inwardly activated, i.e. ready for activity, which means that the sympathetic nervous system is then strongly activated.*  *It can therefore be so beneficial to be carried deeper into relaxation by activating our inherent rest response, the parasympathetic nervous system, and thus create a beneficial balance ... to deepen the pleasant calm ... and to enjoy it.*  *[And the scent of tranquility that envelops you may carry you, help you to gradually allow yourself to be carried into tranquility at your own pace.]*  *Induction*  *An effective way to trigger your body's parasympathetic resting response is to gradually slow down your breathing, which we will now begin.*  *Start by simply focusing your attention on your breathing... Letting the breath come and go ... Inhaling and exhaling.... In ... and ... out ... in ... and ... out ... in ... and ... out ... (pause). And breathing is one of our natural rhythms ... that our body lets happen all by itself ... In ... and ... Out ... In ... and ... Out ... And you can use this rhythm.... To let go of the swaying of your breathing ... of your body .... With every breath ... deeper ... into peace ... let yourself be carried ... in ... and ... out ... in ... and ... out ... (pause)*  *And as your breath comes and goes ... you may allow yourself ... to let the breaths gradually become a little calmer and thus also a little longer ... especially the exhalation ... to become a little longer ... in your rhythm ... at your pace ... just like this ... (pause)*  *And with every exhalation ... every exhalation ... allow yourself to imagine ... that you are exhaling ... letting go ... of everything that might ... still be holding you back ... even deeper ... into calmness ... to let go ... even deeper ... to relax ... (pause) ...*  *Suggestion 1*  *And for many people it helps ... to imagine ... that you want to let go of everything ... inner and outer ... physical ... feelings ... thoughts ... whatever they are ... in bubbles or balloons ... to exhale ... to breathe out ... bubbles ... balloons ... that dissolve ... and fly ... out ... into the wide space ... to let ... dissolve ... to ... let ... go ...*  *And this exhalation ... these bubbles ... balloons ... that dissolve ... allow you ... to feel even freer ... to be carried even deeper ... more relaxed ... into peace ... even deeper. And that has something very liberating about it ... to breathe out ... and to be able to enjoy ... how it already feels ... when you go deeper ... into the silence ...*  *Just take the time ... to enjoy this ... and to let go ... to breathe out ... to let go ... as it is good for you (longer pause)*  *Suggestion 2*  *And while a part of you may already feel more relaxed and calmer, I would like to mentally invite you to a journey into calmness and serenity ... where you may also mentally deepen the calmness and serenity once again.*  *Allow yourself ... to imagine a place of calm, of serenity ... to let yourself mentally ascend ... where you feel calm, serene and at ease ... perhaps this is a place you already know ... from the past, or from the present, perhaps also a place in your imagination ... or a future place ... a place of calm ... of serenity ... of well-being ...*  *Take your time ... and simply see what images ... feelings ... thoughts ... sensations ... arise ... take your time ... let it happen ... just as it suits you ... (pause)*  *And when you have found a place, allow yourself to simply look around ... what you see there ... pictures ... colors ... shapes ... and enjoy ... how good it feels ... in this place of peace ... to simply ... calmly and serenely ... look around ... look at ... the pictures .... To take in the peace ... to connect with it ... to let it carry you ... Take your time ... as it suits you ... (pause).*  *And then allow yourself ... to simply enjoy the sounds ... or the silence ... in this place ... what do you hear there ... enjoy ... connect with the silence ... the sounds ... enjoy ... as it suits you ... (pause)*  *And are there also odors there? ... Sometimes there are smells ... enjoy them ... let yourself be carried away by the pleasant odors ... there in the place of peace ... (pause)*  *Sometimes there is also a flavor? ... savor ... (pause)*  *And then ... how does your body feel there ... in the place of rest ...? Allow yourself ... to savor the pleasant sensations ... to connect ... with the calm ... the pleasant sensations ... your body ... Perhaps there are places that feel particularly good? ... Allow yourself to enjoy these pleasant sensations, the particularly pleasant places ... even let the pleasant sensations spread ... let yourself be carried away ... enjoy ... take your time ... calmness ... serenity ... well-being ... (pause)*  *And with the rhythm of your breathing, allow the well-being, the calm, the serenity, the pleasant sensations to carry you ... allow yourself to simply be ... to enjoy ... in your own way (longer pause)*  *Post-hypnotic suggestion*  *And you can take this calmness, this serenity, this feeling of well-being with you into your waking state, into your everyday life, because your body remembers. And the more often you do this exercise, the easier and more natural it will be for you to activate the calm response, the parasympathetic nervous system ... to enjoy the relaxation and serenity ... so that it becomes increasingly natural for you. [And in everyday life too, the pleasant scent of calm can guide and carry you, remind you and connect you with calm, so that your body remembers and you can increasingly enjoy calm and serenity in everyday life too] (pause)*  *Re-orientation*  *And then you can prepare yourself, very gradually, to orientate yourself back into this space, this time, this place... And to take the pleasant experiences from this relaxation with you into your waking consciousness. And when I count from 0 to 10, to become more awake again with each number, so that you arrive here fresh and awake at 10, fresh and awake...*  *0, 1, 2, 3, 4, 5, 6, 7, 8, 9, 10*  *And stretch and stretch, open your eyes and arrive here fresh and refreshed. The trance is over.* |
|  |
| **Hypnosis Refresher Script** |
| **Used in:** MindSpaceOne Group (From session 2 onwards), Hypnotherapy-Only Group (From session 2 onwards) |
| **Note:** Again, the MindSpaceOne Group version included references to the scent, and these differences were indicated below using [MS1] tags. |
| *Welcome to today's self-hypnosis session.*  *Make yourself comfortable in a quiet and pleasant place where you can let yourself be carried inwards for a while. The aim of this hypnosis is to allow yourself to be carried into a pleasant state of calm and serenity.*  *[Allow yourself to perceive the pleasant aromatic scent of calm and serenity that surrounds you ... savor it. And allow the fragrance of calmness that surrounds you to carry you, to help you to gradually allow yourself to be carried into calmness at your own pace]*  *Begin by focusing your attention on your breathing... Let the breath come and go ... The inhalation and exhalation.... In ... and ... out ... in ... and ... out ... in ... and ... out ... (pause). And breathing is one of our natural rhythms ... that our body lets happen all by itself ... In ... and ... Out ... In ... and ... Out ... And you can use this rhythm.... To let go of the swaying of your breathing ... of your body .... With every breath ... deeper ... into calm ... ... in ... and ... out ... in ... and ... out ... (pause)*  *And then allow yourself ... to go ... to your inner place of calm, of serenity…*  *Take your time ... and just see what images ... feelings ... thoughts ... sensations ... arise ... take your time ... let it happen ... just as it suits you ... (pause) ... And enjoy looking around ... what you see there ... images ... colors ... shapes ... to enjoy ... and to hear.... enjoy the sounds ... or even the silence ... and perhaps also smells ... and a taste ... enjoy. And then ... how does your body feel there ... in the place of peace ...? Allow yourself ... to savor the pleasant sensations ... to connect ... with the calm ... and to enjoy ... take your time ... calm ... serenity ... well-being ... (pause) And let yourself be carried away ... allow yourself to simply be ... to enjoy ... in your own way ...*  *And you can then take this calm, this serenity, this feeling of well-being with you into your waking state, into your everyday life, because your body remembers. And the more often you do this exercise, the easier and more natural it will be for you to activate the calm response ... to enjoy relaxation and serenity ... so that it becomes increasingly natural for you.*  *[And also in everyday life, the pleasant scent of peace can guide and carry you, remind you and connect you with peace, so that your body remembers and you can increasingly enjoy peace and serenity in everyday life as well] (pause)*  *And then you can prepare yourself to orientate yourself back to this space, this time, this place... And to take the pleasant experiences from this hypnosis with you into your waking consciousness. And when I count from 0 to 10, to become more awake again with each number, so that you arrive here fresh and awake at 10, fresh and awake...*  *0, 1, 2, 3, 4, 5, 6, 7, 8, 9, 10*  *And stretch and stretch, open your eyes and arrive here fresh and refreshed. The trance is over.* |
|  |
| **Minimal-Intervention Pause Script** |
| **Used in:** Aromatherapy-Only (Olfactory Cue and Minimal-Intervention Pause), Control Group (Minimal-Intervention Pause) |
| *Give yourself a break, make yourself comfortable, close your eyes if you like, and take a few minutes to absorb the scent and let it work*  *(5-minute break)*  *And then allow yourself to complete this exercise.* |

| **Supplementary Material 4. Descriptive Statistics.** | | | | | |
| --- | --- | --- | --- | --- | --- |
| **Visit** | **Variable** | **MindSpaceOne**  (Mean ± SD, range) | **Hypnotherapy-only**  (Mean ± SD, range) | **Aromatherapy-only**  (Mean ± SD, range) | **Control**  (Mean ± SD, range) |
| V0 | MDMQ total score | 74.52 ± 18.23, 37-111 | 69.17 ± 16.03, 37-99 | 73.56 ± 15.32, 42-107 | 69.91 ± 18.07, 35-109 |
|  | MDMQ good mood-bad mood | 27.61 ± 6.31, 14-40 | 25.94 ± 5.93, 14-38 | 27.58 ± 6.22, 14-40 | 25.67 ± 6.83, 10-39 |
|  | MDMQ calmness-restlessness | 24.11 ± 7.27, 9-40 | 21.81 ± 6.14, 10-34 | 23.73 ± 5.62, 12-36 | 23.15 ± 6.53, 11-38 |
|  | MDMQ alertness-tiredness | 22.80 ± 6.50, 8-39 | 21.41 ± 5.77, 8-32 | 22.25 ± 5.41, 11-33 | 21.09 ± 6.35, 10-37 |
|  | PSS Helplessness | 17.56 ± 4.85, 7-28 | 18.89 ± 4.18, 10-28 | 17.77 ± 4.14, 9-27 | 18.69 ± 4.76, 10-27 |
|  | PSS self-efficacy | 10.62 ± 2.57, 4-16 | 11.67 ± 2.49, 4-18 | 10.85 ± 2.75, 5-18 | 11.76 ± 2.65, 4-17 |
|  | PSS total | 28.18 ± 6.86, 14-41 | 30.56 ± 6.12, 15-46 | 28.62 ± 6.34, 15-45 | 30.44 ± 6.75, 15-42 |
|  | WHO 5 | 18.67 ± 5.14, 9-28 | 18.96 ± 4.61, 11-26 | 18.49 ± 4.85, 9-28 | 19.56 ± 5.01, 8-28 |
| V1 | MDMQ total score | 85.16 ± 15.37, 48-112 | 80.45 ± 14.90, 43-114 | 78.38 ± 15.19, 44-112 | 77.33 ± 16.30, 24-111 |
|  | MDMQ good mood-bad mood | 30.38 ± 5.59, 13-40 | 28.99 ± 5.46, 18-40 | 28.71 ± 5.49, 14-40 | 27.85 ± 6.09, 8-40 |
|  | MDMQ calmness-restlessness | 27.99 ± 5.82, 10-37 | 26.41 ± 5.96, 11-40 | 25.80 ± 5.76, 11-39 | 25.57 ± 5.92, 8-40 |
|  | MDMQ alertness-tiredness | 26.79 ± 5.43, 16-38 | 25.05 ± 5.04, 13-37 | 23.88 ± 5.65, 11-38 | 23.91 ± 5.72, 8-38 |
|  | PSS Helplessness | 15.84 ± 4.45, 8-26 | 16.49 ± 4.34, 7-26 | 16.30 ± 4.35, 8-26 | 16.91 ± 4.43, 7-26 |
|  | PSS self-efficacy | 9.67 ± 2.36, 4-15 | 10.45 ± 2.80, 4-17 | 10.57 ± 2.57, 5-17 | 10.69 ± 2.77, 4-17 |
|  | PSS total | 25.51 ± 6.43, 13-40 | 26.95 ± 6.57, 11-41 | 26.87 ± 6.33, 14-43 | 27.60 ± 6.49, 11-42 |
|  | WHO 5 | 15.60 ± 4.78, 8-26 | 16.36 ± 4.52, 8-27 | 17.27 ± 4.39, 10-26 | 17.64 ± 5.06, 6-30 |
| V2 | MDMQ total score | 87.88 ± 16.31, 35-118 | 85.04 ± 14.28, 57-113 | 83.94 ± 14.82, 51-115 | 79.95 ± 16.67, 24-120 |
|  | MDMQ good mood-bad mood | 31.15 ± 5.37, 13-40 | 30.13 ± 5.45, 19-40 | 30.08 ± 5.13, 16-40 | 29.26 ± 6.06, 8-40 |
|  | MDMQ calmness-restlessness | 28.96 ± 6.07, 10-40 | 28.02 ± 5.53, 10-40 | 27.62 ± 5.69, 10-37 | 26.16 ± 5.96, 8-40 |
|  | MDMQ alertness-tiredness | 27.77 ± 6.19, 11-39 | 26.89 ± 4.87, 15-39 | 26.23 ± 5.60, 10-38 | 24.52 ± 6.06, 8-40 |
|  | PSS Helplessness | 14.48 ± 4.36, 6-28 | 14.96 ± 4.23, 7-27 | 14.94 ± 4.44, 7-26 | 15.71 ± 4.70, 6-30 |
|  | PSS self-efficacy | 9.58 ± 2.74, 4-16 | 9.89 ± 2.70, 4-17 | 9.68 ± 2.56, 4-15 | 10.24 ± 2.63, 4-16 |
|  | PSS total | 24.06 ± 6.53, 11-44 | 24.84 ± 6.26, 12-41 | 24.62 ± 6.56, 12-39 | 25.96 ± 6.66, 10-44 |
|  | WHO 5 | 14.54 ± 4.52, 7-27 | 15.17 ± 4.60, 8-25 | 15.85 ± 4.73, 7-27 | 16.51 ± 4.93, 6-30 |
| V3 | MDMQ total score | 86.52 ± 15.64, 54-120 | / | 84.94 ± 15.71, 36-118 | / |
|  | MDMQ good mood-bad mood | 30.66 ± 5.21, 18-40 | / | 30.50 ± 5.46, 12-40 | / |
|  | MDMQ calmness-restlessness | 28.28 ± 6.01, 14-40 | / | 27.86 ± 5.82, 13-39 | / |
|  | MDMQ alertness-tiredness | 27.58 ± 5.90, 14-40 | / | 26.59 ± 5.96, 8-39 | / |
|  | PSS Helplessness | 15.00 ± 4.30, 6-25 | / | 14.57 ± 4.44, 6-26 | / |
|  | PSS self-efficacy | 9.53 ± 2.42, 4-16 | / | 9.74 ± 2.66, 4-16 | / |
|  | PSS total | 24.53 ± 6.24, 10-39 | / | 24.31 ± 6.53, 11-40 | / |
|  | WHO 5 | 15.07 ± 4.81, 5-25 | / | 15.39 ± 4.83, 5-27 | / |
| SD, standard deviation; V0, visit 0 (baseline); MDMQ, Multidimensional Mood State Questionnaire; PSS, Perceived Stress Scale; WHO 5; V1, visit 1 (2 weeks after baseline); V2, visit 2 (4 weeks after baseline); V3, visit 3 (5 weeks after baseline).  Note: Groups hypnotherapy-only and control were discontinued at V2 because V3 was assessed for classical conditioning effects. | | | | | |

| **Supplementary Material 5. Within-group change from baseline to post-intervention.** | | | | |
| --- | --- | --- | --- | --- |
| **Outcome** | **Mean Change (V2 – V0)** | **95% CI** | | **p value** |
|  |  | **Lower** | **Upper** |  |
| ***Primary outcome: MDMQ calmness-restlessness (ITT)*** | | | | |
| MindSpaceOne | 4.543 | 3.104 | 5.981 | < 0.001 |
| Hypnotherapy-only | 5.145 | 3.717 | 6.572 | < 0.001 |
| Control | 3.112 | 1.894 | 4.330 | < 0.001 |
| V2, visit 2 (4 weeks post intervention); V0, visit 2 (baseline); CI, Confidence interval; MDMQ, Multidimensional Mood State Questionnaire; ITT, Intention-to-treat | | | | |

| **Supplementary Material 6. Primary and Secondary Outcomes (PP, n = 383)** | | | | | | |
| --- | --- | --- | --- | --- | --- | --- |
| **Outcome** | **Estimate (β)** | **95% CI** | | **p value** | **Adjusted p value** | **Cohen’s d** |
|  |  | **Lower** | **Upper** |  |  |  |
| ***Primary outcome: MDMQ calmness-restlessness*** | | | | | | |
| MindSpaceOne vs. control | 2.589 | 1.08 | 4.10 | **0.0009** | **0.007** | 0.49 |
| Hypnotherapy-only vs. control | 2.250 | 0.71 | 3.79 | **0.005** | **0.016** | 0.43 |
| ***Secondary outcomes*** | | | | | | |
| ***MDMQ alertness-tiredness*** | | | | | | |
| MindSpaceOne vs. control | 2.699 | 1.18 | 4.21 | **0.0006** | **0.007** | 0.51 |
| Hypnotherapy-only vs. control | 2.320 | 0.78 | 3.86 | **0.003** | **0.012** | 0.44 |
| ***MDMQ good mood-bad mood*** | | | | | | |
| MindSpaceOne vs. control | 1.257 | -0.08 | 2.60 | 0.067 | 0.119 | 0.27 |
| Hypnotherapy-only vs. control | 0.833 | -0.53 | 2.20 | 0.233 | 0.287 | 0.18 |
| ***MDMQ total*** | | | | | | |
| MindSpaceOne vs. control | 6.446 | 2.47 | 10.43 | **0.002** | **0.011** | 0.46 |
| Hypnotherapy-only vs. control | 5.423 | 1.37 | 9.48 | **0.009** | **0.023** | 0.39 |
| ***PSS-10 total*** | | | | | | |
| MindSpaceOne vs. control | -0.555 | -2.07 | 0.96 | 0.474 | 0.542 | 0.11 |
| Hypnotherapy-only vs. control | -0.979 | -2.51 | 0.56 | 0.212 | 0.287 | 0.19 |
| ***PSS-10 helplessness*** | | | | | | |
| MindSpaceOne vs. control | -0.648 | -1.70 | 0.41 | 0.230 | 0.287 | 0.18 |
| Hypnotherapy-only vs. control | -0.837 | -1.91 | 0.24 | 0.127 | 0.203 | 0.23 |
| ***PSS-10 self-efficacy*** | | | | | | |
| MindSpaceOne vs. control | -0.453 | -0.73 | 0.64 | 0.896 | 0.896 | 0.02 |
| Hypnotherapy-only vs. control | -0.159 | -0.84 | 0.53 | 0.650 | 0.693 | 0.07 |
| ***WHO 5*** | | | | | | |
| MindSpaceOne vs. control | -1.49 | -2.62 | -0.36 | **0.010** | **0.023** | 0.38 |
| Hypnotherapy-only vs. control | -1.09 | -2.25 | 0.06 | 0.064 | 0.119 | 0.28 |
| ITT, Intention-to-treat; CI, Confidence interval; MDMQ, Multidimensional Mood State Questionnaire; PSS-10, Perceived Stress Scale 10 items | | | | | | |

| **Supplementary Material 7. Follow-up: Conditioning Effects** | | | | | |
| --- | --- | --- | --- | --- | --- |
| **Outcome** | **Estimate (β)** | **95% CI** | | **p value** | **Cohen’s d** |
|  |  | **Lower** | **Upper** |  |  |
| ***Primary outcome: MDMQ calmness-restlessness (ITT)*** | | | | | |
| MindSpaceOne vs. aromatherapy-only | -0.372 | -1.66 | 0.91 | 0.570 | 0.08 |
| ***Primary outcome: MDMQ calmness-restlessness (PP)*** | | | | | |
| MindSpaceOne vs. aromatherapy-only | -0.461 | -1.49 | 0.57 | 0.381 | 0.13 |
| CI, Confidence interval; MDMQ, Multidimensional Mood State Questionnaire; ITT, Intention-to-treat; PP, Per-Protocol | | | | | |

| **Supplementary Material 8. Follow-up: Conditioning Effects on Olfactory Preference Subgroups** | | | | | |
| --- | --- | --- | --- | --- | --- |
| **Outcome** | **Estimate (β)** | **95% CI** | | **p value** | **Cohen’s d** |
|  |  | **Lower** | **Upper** |  |  |
| ***Primary outcome: MDMQ calmness-restlessness (PP)*** | | | | | |
| MindSpaceOne vs. aromatherapy-only | -0.433 | -1.94 | 1.07 | 0.42 | 0.12 |
| CI, Confidence interval; Multidimensional Mood State Questionnaire; PP, Per-Protocol | | | | | |

**
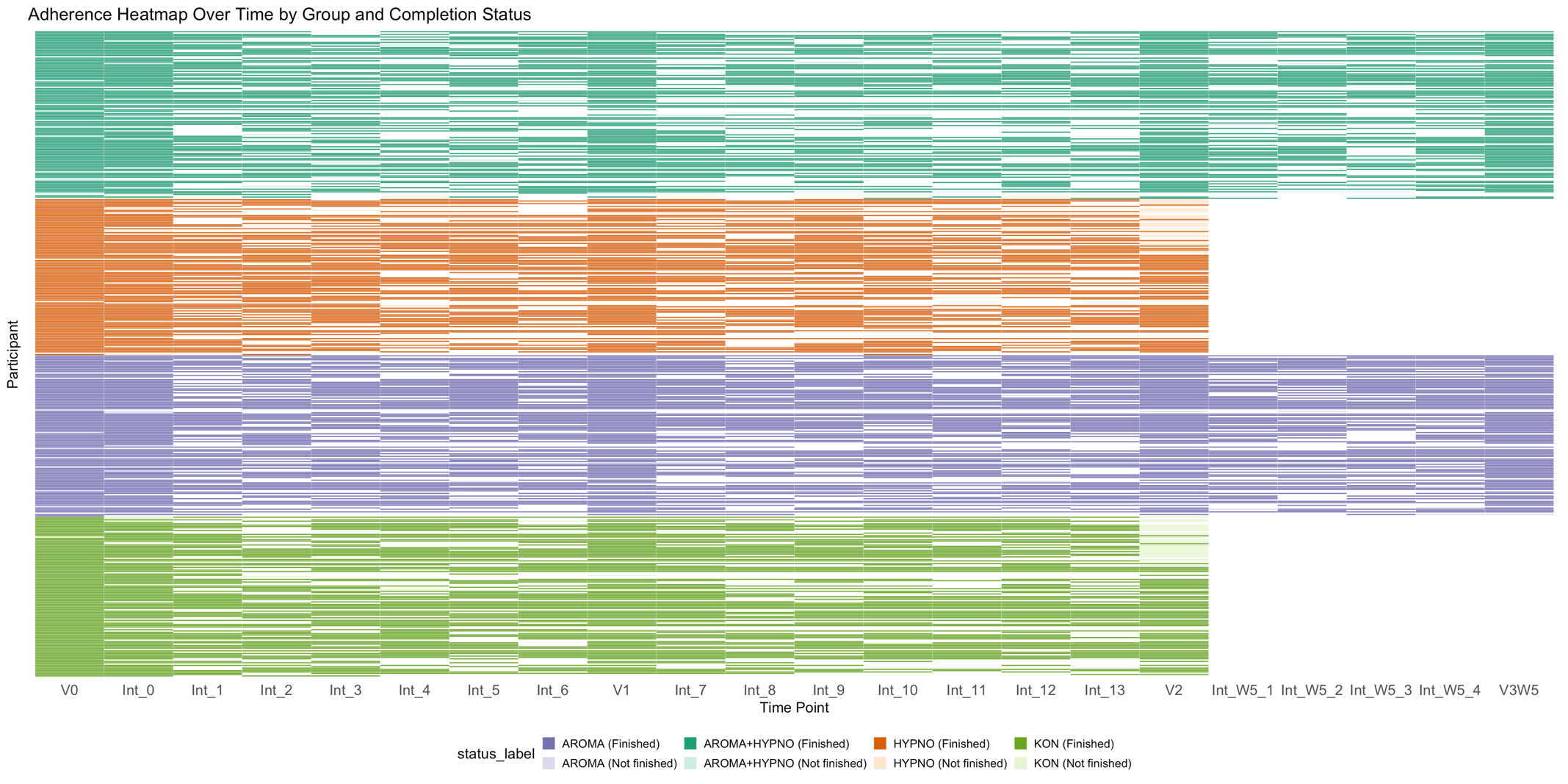
**

**Supplementary Material 9. Adherence Heatmap Over Time by Group and Completion Status.** Each row represents one participant, and each column a scheduled session (Int_0 to Int_W5_4) or study visit (V0 to V3). Tiles are color-coded based on intervention group and completion status of each session (finished vs. not finished). The intensity of color reflects whether the session as completed (solid color) or not (pale). Participants are grouped and ordered by intervention arm (top to bottom): MindSpaceOne (green), Hypnotherapy-only (orange), Aromatherapy-only (purple), and Control (light green). The figure illustrates overall adherence patterns over time, with slightly lower completion rates in the hypnotherapy-based groups.

| **Supplementary Material 10. Adverse events categories** | |
| --- | --- |
| **Category** | **Answers, n** |
| External life events | 19 |
| Unspecific/ unclear | 16 |
| Physical health complaints | 13 |
| Intervention-related positive | 10 |
| Intervention-related negative | 4 |
| Psychological burden | 3 |
| Technical/ organizational issues | 3 |
| Pet- or household-related issues | 1 |
