# Supplementary material for: Promoting relaxation through essential oil-enhanced digital hypnotherapy: A randomized controlled trial
Source: Psychol Med. 2026 Mar 30;56:e80. doi: 10.1017/S0033291726103778 (PMC13079237; doi:10.1017/S0033291726103778)
Supplement: Ngandeu Schepanski et al. supplementary material 2 — Ngandeu Schepanski et al. supplementary material [file S0033291726103778sup002.pdf]

Transparent Reporting for Essential Oil & Aroma Therapeutic Studies (TREATS) Checklist

In addition to conventional research quality appraisal tools, research involving essential oils and aromatherapy should meet requirements for validity and reliability. Complete reporting facilitates study replication and progression for programs of research. For clarity, use the companion TREATS explanatory document during this critique process.

AROMATIC RESEARCH QUALITY APPRAISAL TASKFORCE
ARQAT

As you fill in the tool, we suggest you use X's. The columns are designed to show the value of the X. So, in the first column, an X = 1, fully met. And in the middle column, X = 0.5 or ½ a point. And, finally, in that last column, the X = 0 points. The explanation and comment section is there for you to write those things that might help you remember 'why' you thought that X went in that column. When used as a critique tool, these comments serve as feedback to the researcher.

Reviewer:
Article reviewed:
Delivery method: Topical ____ Inhalation _X_ Both ____

	Category (see Explanatory document for more details of each item)	Met =1 or *N/A	Partially met = 0.5 (Explain)	Not Met = 0	Explanations/ Comments/ Questions
**Section 1: Essential oils (EO)**					
1	Essential oil (EO) binomial (botanical) name (*Genus species)*	X			Supplementary material 1
2	Production method	X			Steam distillation of twigs
3	Plant part	X			Twigs explicitly stated
4	Cultivation Method	X			Certified organic cultivation
5	Country of Origin	X			Austria
6	Source	X			Manufacturer: PRIMAVERA LIFE GmbH
7	Batch number of the EO	X			Batch # A3514(3)
8	Identification of plant constituents	X			GC profile with constituent percentages provided
	**Total Section 1 (possible points = 8)**	8/8			
**Section 2A: Topical Application- Complete ONLY if topical delivery method used**					
1	Dilution of EO (if applicable)				
2	Dose of EO				
3	Body surface area EO contacts				
4	Frequency of EO				
5	Duration of EO				
6	Description of control or placebo				
7	Carrier(s) name, including full binomial				
8	Source of carrier or delivery system				
	**Total Section 2A (possible points = 8)**				
**Section 2B: Inhalation- Complete ONLY if inhalation delivery method used**					
1	Mode of inhalation	X			Indirect/ambient inhalation via room spray
2	Dose of EO	X			Number of spray specified thereby volume
3	Frequency of EO	X			Every 2nd day over intervention period

cc BY NC ND

Category (see Explanatory document for more details of each item)		Met =1 or *N/A	Partially met = 0.5 (Explain)	Not Met = 0	Explanations/ Comments/ Questions
4	Duration of EO	X			4-week intervention (+ followup)
5	Description of control or placebo	X			Control condition without olfactory stimulus
6	Carrier(s) name, including full binomial<br>*Mark **N/A** if no carrier used	X			Ethanol and water reported
7	Source of carrier or delivery system	X			Commerical spray product
	**Total Section 2B (possible points = 7)**	7/7			
**Section 3: Aromatic Intervention**					
1	A clear description of aromatherapy interventions, outcome measures, and adherence to the researcher's protocol<br><br>Give **partial credit** if the description of intervention & outcome measures is cited but not clear enough to replicate, or the research protocols were not followed	X			Intervention protocol, timing, adherence described
2	The rationale for EO(s)	X			Forest-associated oil selected a priori
3	Theoretical or conceptual framework	X			Conditioning framework + restorative nature theory
4	Professional aromatherapist consulted			X	No aromatherapist involved, self-administered
5	Safety considerations		X		Adverse event monitoring reported
6	Report of allergic and adverse reactions	X			Adverse events monitored and reported
7	Safety consideration of EO storage during trial			X	Storage not specified
	**Total Section 3 (possible points = 7)**	4.5/7			
**Section 4a: Olfactory function questions** (Asked prior to trial)					
1	Anosmia			X	Not assessed
2	Previous use of EOs	X			Prior aromatherapy use assessed at baseline
	**Total Section 4a (possible points = 2)**	1/2			
**Section 4b: Olfactory bias questions if practical in an experimental setting** (Asked as part of the trial. If not asked, the researcher may give an explanation for excluding these steps. Give partial credit if mentioned in the limitations section).					
1	Olfactory testing			X	Home-based setting; not controlled
2	Odor recognition testing			X	Not assessed
3	Participants' expectations stated			X	Not assessed
4	Odor preference bias	X			Valence ratings collected and analyzed
5	Perceived aroma intensity			X	Not assessed
6	Any adverse effect from olfaction testing	N/A			No olfactory testing performed
	**Total Section 4b (possible points = 6)**	1/6			

**Please transfer points from each section in the table below. Add points to obtain total.

CC BY NC ND

*FOR N/A—when NA is used to describe the non-applicability of something within this tool, that should be taken out of the calculation and the total number changed (e.g., 16/29 if NA used for 2B: 6 Carrier(s) name and using the INHALATION only pathway).

Section (Points)	Total section points INHALATION	Total section points TOPICAL	Total section points Topical AND Inhalation
Section 1 (8)	8/8	/8	/8
Section 2 (7-15)	7/7	/8	/15
Section 3 (7)	4.5/7	/7	/7
Section 4a (2)	1 /2	/2	/2
Section 4b (6)	1 /6	/6	/6
Total (30-38) Poor, Fair, Good	21.5/30	/31	/38

0-10 = Poor; 11-20 = Fair; 21-38 = Good

(These ratings serve as guidance. Additional comments & conclusions are necessary to qualify these ratings. For example: "16/30—Fair quality for aromatherapy practice in this study with acknowledgment of best practice observed for essential oil reporting and safety. No mention of how olfactory bias or function was accounted for."

ADDITIONAL REVIEWER COMMENTS:

cc BY NC ND
